# Preparation of Organic-Inorganic Phosphorus-Nitrogen-Based Flame Retardants and Their Application to Plywood

**DOI:** 10.3390/polym15143112

**Published:** 2023-07-21

**Authors:** Chao Deng, Yu Ji, Meng Zhu, Yuqing Liang, Hao Jian, Zhichun Yan, Mingyu Wen, Heejun Park

**Affiliations:** 1 Department of Wood Material Science and Engineering Key Laboratory, College of Materials Science and Engineering, Beihua University, Jilin 132013, China; jlbhdengchao@163.com (C.D.); xyjiyu@163.com (Y.J.); wugengyeliuli@163.com (M.Z.); 17354371453@163.com (Y.L.); jianhao_1999@163.com (H.J.); 2 Jilin Xing Yuan Machinery Technology Co., Jilin 132000, China; 13843215085@163.com; 3 Department of Housing Environmental Design, Research Institute of Human Ecology, College of Human Ecology, Jeonbuk National University, Jeonbuk 54896, Republic of Korea

**Keywords:** synergistic effect, flame-retardant plywood, phosphorus-nitrogen flame retardants, laminated double hydroxide

## Abstract

The combustibility of wood can be improved by physical and chemical means, thus expanding the use of wood products. In this study, two novel phosphorus-nitrogen flame retardants (UCPR and MCPR) were developed, and the intercalated modified layered double hydroxides (LDH) thereof were designated as UCPR-LDH and MCPR-LDH. By impregnating poplar veneer with UCPR-LDH and MCPR-LDH solutions, the study investigated the effects of different concentrations (1%, 5%, 10%), processes (vacuum-pressure impregnation, room temperature impregnation, normal-pressure impregnation), and impregnation times (2 h, 3 h, 24 h, 48 h) on the weight-gain rate of veneer. The optimal process was then selected for preparing formaldehyde-free three-layer plywood. Nuclear magnetic resonance (NMR) and Fourier transform infrared (FTIR) were used to characterize the UCPR and MCPR. Meanwhile, gel-permeation chromatography (GPC) demonstrated that the molecular weight of the synthesized flame retardants increased as their molecular distribution became more uniform. The impregnation process was performed at normal temperature and pressure for 48 h at a 5% flame retardant concentration. Results from cone calorimetry indicate that the UCPR-LDH plywood exhibits a peak heat release rate that is 30.43% lower than that of the control group, demonstrating superior thermal barrier performance. The smoke emission of the MCPR-LDH plywood was reduced by 33.62% compared to the control group, indicating superior smoke suppression performance. This method presents a viable approach for synthesizing organic-inorganic flame retardants.

## 1. Introduction

Fire is one of the most common disasters and poses a great threat to people’s lives, property, and public safety [1,2]. Most of the polymers that we come into contact with in our lives, such as wood, polyurethane, plastic, etc., are combustible [3,4,5,6,7,8]. Wood, one of the world’s most commonly used raw materials, not only has naturally beautiful grain and color, but is renewable, easy to process, plays the role of regulating the temperature and humidity environment, and provides a comfortable sensory experience when used in furniture and building materials [9,10,11,12]. Therefore, wood is widely employed in the construction, manufacturing, packaging, decorative, and transportation industries [13,14]. However, wood is a complex porous natural organic substance made of cellulose, hemicellulose, and lignin, and has inherent flaws such as low strength, flimsiness, high variability, flammability, and susceptibility to mold, which limit its use in specific applications [15,16]. To minimize potential fire hazards, there is an urgent need for fire-retardant treatment of wood to increase fire safety and broaden the spectrum of use.

The process of burning wood is quite complicated and includes both physical and chemical processes [17]. Wood that has been physically or chemically modified may effectively interrupt the wood-burning cycle. Current wood flame-retardant treatment techniques include impregnation, surface modification, sol-gel, high-temperature heat treatment, etc. [18,19,20]. Meanwhile, various flame retardants can be employed for wood treatment, since halogen retardants pose risks to both human health and the environment upon combustion. Therefore, more eco-friendly alternatives such as phosphorus-, nitrogen-, intumescent- and inorganic-based flame retardants are recommended. The selection of different techniques and the type of flame retardant used for flame-retardant treatment can effectively modify the flammability characteristics of wood [21,22,23]. Fan et al. [24] partially delignified natural wood, impregnated ammonium dihydrogen phosphate into the cell walls of the wood at atmospheric pressure and densified the wood by cyclically alternating hot pressing to produce a functional wood with desirable mechanical and fire-retardant properties. Zhong et al. [25] synthesized a new epoxy flame retardant from eugenol by nucleophilic substitution and Prilezhaev epoxidation and applied it to wood-surface finishing in combination with a curing agent. The results demonstrated that the surface-modified coating possessed flame-retardant properties. Li et al. [26] impregnated a biocompatible and mineral-inducing gelatin into wood and used the sol-gel technique to mineralize silica in wood pores and cell cavities in situ, resulting in mineralized wood composites with good flame-retardant and mechanical qualities.

Because of their low smoke emission, excellent thermal stability, and strong flame-retardant qualities, phosphorus-nitrogen flame retardants (P-N FRs) are extensively employed in wood flame retardants. Phosphorus-nitrogen compounds have a synergistic effect and can take advantage of both phosphorus and nitrogen flame retardants. The primary role of these compounds is dehydration and carbonization, and at high temperatures they thermally break down into polyphosphoric acid, accompanied by the release of non-combustible gas, which aids in the formation of an expanded carbon layer [27,28,29,30]. Zhang et al. [31] prepared an ammonium-phytate-based flame retardant using the reaction of phytic acid and urea and impregnated it into Loblolly pine (*Pinus taeda*) wood. The total heat release and smoke release of the treated wood decreased by 81.5% and 43.2%, respectively, compared to untreated wood, and an increased graphitized char layer was created that effectively restricted the transmission of heat and combustible gases. To lower the flammability of plywood, Kolibaba et al. [32] introduced a flame-retardant polyelectrolyte complex coating after photopolymerization. A combination of polyethyleneimine and hydroxyethyl methacrylate phosphate was used to produce the coating on the plywood surface. The peak heat release rate and total smoke release of the flame-retardant plywood were reduced by 13% and 56%, respectively, when the coating was added in an amount of only 1.6% by weight of plywood.

Furthermore, researchers often use inorganic compounds to assist P-N FRs to enhance the flame-retardant effect and give the substrate versatility [33,34,35]. Layered double hydroxide (LDH) is a novel two-dimensional inorganic nanomaterial. Due to the weak interaction between the anions in the middle of the layered material and between the layers, they can usually be exchanged for intercalation modification. And because LDH has cooling, dilution, isolation and smoke suppression effects at high temperatures, it can be applied to flame-retardant fields to exert desirable effects. Zhang et al. [36] selected Mg/Al-LDH to modify guanidine phosphate to obtain a more complete two-dimensional lamellar structure with suitable grain size and modified the cellulose composites. The thermal stability of the modified fibrous material was greatly enhanced, and the ultimate oxygen index was increased to 27.7% with an addition of 15 wt%. Jeevananthan et al. [37] employed cyclic trinitrate carboxylates intercalated in Mg/Al-LDH for epoxy-coated wood. The results showed that the ultimate oxygen index could reach 29% and the vertical combustion test could reach the V-0 class. In addition, Mg/Al-LDH and cyclotrizonitrile exerted an excellent synergistic flame-retardant effect, forming a nonvolatile protective layer on the surface and isolating it from the air.

Pyrovatex CP (CP) is a reagent containing P and N elements, i.e., N-hydroxymethyl-3-dimethoxyphosphoryl propionamide. It is mainly used for flame retardance of fibers and cotton fabric and is a highly durable and efficient flame-retardant reagent. In this study, the optimal reaction process of CP with urea or melamine was investigated, and two novel P-N FRs were prepared by controlling the three factors of molar ratio of CP to urea or melamine, reaction temperature, and reaction time. UCPR and MCPR were intercalated in Mg/Al-LDH to achieve an organic-inorganic flame retardant. The two novel P-N FRs were characterized and analyzed by Fourier transform infrared (FTIR) and gel permeation chromatography (GPC). The flame retardant was used to impregnate poplar veneer and modified isocyanate API adhesive and pressed into three-layer flame retardant plywood. Cone calorimetry was used to evaluate the plywood’s flame-retardant and smoke suppression capabilities. In addition, the residual carbon of the cone calorimetry was characterized using FTIR for functional group analysis. This study provides a simple method for the preparation of flame-retardant wood materials.

## 2. Materials and Methods

### 2.1. Materials

Polyvinyl alcohol (type 1788) and P-MDI were purchased from Tianjin Beichen Founder Reagent Co. (Tianjin, China). Pyrovatex CP was purchased from Ciba (Basel, Switzerland). Urea, melamine, anhydrous sodium carbonate, anhydrous magnesium sulfate, anhydrous aluminum sulfate, sodium hydroxide, and hydrochloric acid were procured from Tianjin Damao Chemical Reagent Co. (Tianjin, China). Calcium carbonate and calcium chloride were purchased from Tianjin Yongda Chemical Reagent Co. (Tianjin, China), while the vinyl acetate-ethylene copolymer emulsion was supplied by Zhengzhou Hongbaili Chemical Products Co. (Zhengzhou, China). The poplar veneer originated from Dunhua, Jilin, and deionized water was prepared in-house.

### 2.2. Preparation of UCPR and MCPR

**Scheme 1:**
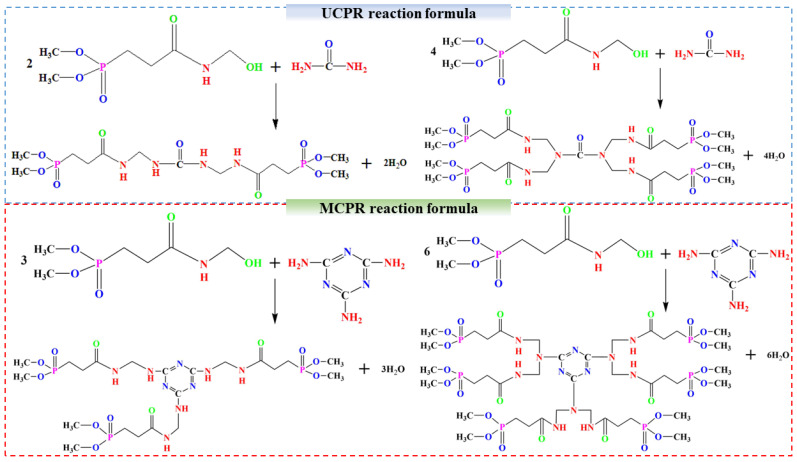
The synthetic route of UCPR and MCPR.

The chemical reagents employed in this study were of analytical grade and utilized without any additional purification. In a typical synthesis, CP (40 g) was dissolved in 9.23 g of deionized water to form solution 1. Solution 1 was added dropwise to a three-mouth flask, 1 g of urea was added, and the mixture was heated at 75 °C. When the temperature reached 75 °C, the mixture was subjected to rapid stirring. After reacting for 30 min, another 1 g of urea was added, the reaction was continued for 30 min, and finally the mixture was stirred and reacted for 15 min to fully react the urea. After the reaction was completed, the heat was turned off, and the mixture was subjected to slow stirring to cool it to room temperature and then poured out and stored in a sealed container. The specific process of the synthesis is shown in Table 1, and the possible synthesis schemes are shown in Scheme 1.

The preparation of MCPR was performed using the UCPR preparation method. First, CP (37.67 g) was dissolved in 8.693 g of water while stirring to form solution 2. Then, 0.5 g of melamine was added to a three-mouth flask with 46.363 g of solution 2 and kept at 70 °C for 15 min, and then 0.5 g of melamine was added dropwise while stirring for another 15 min. Finally, melamine was added twice, and allowed to react each time for 15 min. After the reaction was completed, the mixture was cooled to room temperature, taken out, and stored in a sealed container. The specific process of the synthesis is shown in Table 2, and the possible synthesis schemes are shown in Scheme 1.

### 2.3. Preparation of UCPR-LDH and MCPR-LDH

A mixture of 7.23 g of MgSO_4_, 10 g of Al_2_(SO_4_)_3_ and 50 mL of distilled water was placed in a beaker, stirred until completely dissolved, and recorded as liquid A. A mixture of 7.2 g of sodium hydroxide, 1.91 g anhydrous sodium sulfate, and 50 mL of deionized water was placed in a beaker, stirred until completely dissolved, and recorded as liquid B. Then, 4 g of UCPR or 8 g of MCPR was placed in a three-necked flask, 50 mL of deionized water was added, and the mixture was stirred evenly and heated to 75 °C. The stirring speed was increased, and and equal amounts of liquid A and liquid B were slowly added to the three-mouth flask and stirring was continued for 2 h after drop addition. At the conclusion of the reaction, the reactants were subjected to aging at 75 °C for a duration of 30 min. Following this, they were filtered and washed with distilled water until reaching a pH level of 7. Finally, the resulting products were dried at a temperature of 80 °C in order to obtain white powder UCPR-LDH or MCPR-LDH.

### 2.4. Preparation of Three-Layer Plywood

The poplar veneer was baked to absolute dryness at 60 °C, placed in a 1% sodium hydroxide solution, and drained. To identify the optimal procedure, the poplar veneer was impregnated with various concentrations under vacuum or normal temperature and varying pressure circumstances, and the veneer was dried after impregnation to create an impregnated flame-retardant poplar veneer. The aqueous polymer isocyanate (API) adhesive was prepared as described by Wen et al. [38]. Calcium carbonate, UCPR-LDH, and MCPR-LDH were used as inorganic fillers, corresponding to the control, UCPR-LDH, and MCPR-LDH plywood, respectively. The API adhesive and poplar veneer were assembled into three-layer plywood and hot-pressed with a hot-press temperature of 110 °C, a hot-press time of 3 min, and a hot-press pressure of 1.2 MPa.

### 2.5. Characterization

The samples were dried under vacuum until a constant weight was achieved, ground into a fine powder form, analyzed using an IRAffinity-1S Fourier transform infrared spectrometer with a sample-to-potassium bromide mass ratio of 1:100, and then pressed into tablets for infrared scanning. The selected spectral range was 4000–400 cm^−1^ with 32 scans and a resolution of 4 cm^−1^.

Nuclear magnetic resonance (NMR) spectra ^13^C-NMR and ^31^P-NMR spectra were recorded in Fourier transform mode on a Bruker Avance III 400 MHz NMR spectrometer. The solvent was D_2_O.

The CP, UCPR, and MCPR samples were vacuum-dried to a constant weight and crushed into powder. The GPC instrument model was PL-GPC 50, the column was PLgel MIXED-B LS, and the liquid mobile phase was N,N-Dimethylformamide (DMF) at a flow rate of 1 mL/min and a temperature of 40 °C. The samples were analyzed for molecular weight and dispersion using gel permeation chromatography (GPC).

Poplar veneer samples with dimensions of 100 mm × 100 mm × 1.5 mm were placed in an oven at 60 °C and baked to a constant weight. The dried poplar veneer was soaked in the 1% NaOH solution for 2 h before being drained and placed in LDH suspension, where the concentration was adjusted to 1%, 5%, or 10% for 24 or 48 h at room temperature and pressure. Vacuum-pressure impregnation was used as a comparative process at an LDH suspension concentration of 10%, maintained under vacuum for 30 min, and then pressurized to 1.2 MPa for 2 h or 3 h and dried to constant weight. Comparative testing was used to evaluate the best experimental methods for UCPR-LDH and MCPR-LDH. During the impregnation process, the weight-gain rate of poplar veneer indicated the adherence and precipitation of flame retardant solution in the veneer. The treated veneer’s weight-gain rate (WGR) was estimated using Equation (1):

(1) $$\mathrm{WGR}=\frac{m_{1}{-m}_{0}}{m_{0}}\times 100\%$$

where m_0_ is the absolute mass of the sample before immersion, g, and m_1_ is the absolute mass of the sample after immersion, g.

Samples with dimensions of 100 mm × 100 mm × 1.5 mm were pressed into three-layer plywood. The test was performed in accordance with ISO 5660-1:2015 standards, with a thermal radiation power of 50 kW·m^−2^, a temperature of 800 °C, and a test period of 400–600 s, and vertical flame heating of the sample. The following data were recorded during the combustion process: time to ignite (TTI, s), total heat release (THR, MJ/m^2^), heat release rate (HRR, kW/m^2^), peak heat release rate (pHRR, kW/m^2^), peak CO production rate (pCOPR, g/s), peak CO_2_ production rate (pCO_2_PR, g/s), mass loss rate (MLR, g/s), mass ratio (MR), total smoke production (TSP, m^2^), smoke production rate (SPR, m^2^/s) and peak smoke production rate (pSPR, m^2^/s).

## 3. Results and Discussion

### 3.1. FTIR

**Figure 1:**
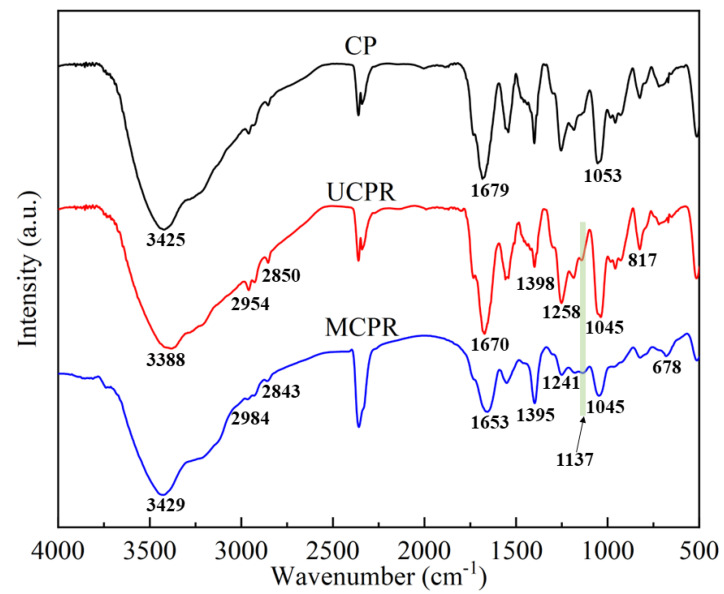
FTIR spectra of CP, UCPR, and MCPR.

The infrared spectra of Pyrovatex CP, UCPR, and MCPR are shown in Figure 1. According to the infrared spectra, it can be seen that the reaction of urea or melamine with CP did not change the main structure of the CP molecular chain. The UCPR has antisymmetric and symmetric stretching vibrational peaks of CH_2_ at 2954 cm^−1^ and 2850 cm^−1^. The MCPR has antisymmetric and symmetric stretching vibrational peaks of CH_2_ at 2984 cm^−1^ and 2843 cm^−1^. The stretching vibration peaks of C=O bonds of UCPR and MCPR are 1670 cm^−1^ and 1653 cm^−1^, respectively. The symmetric variable angle vibration peaks of −CH_2_ are 1398 cm^−1^ and 1395 cm^−1^, respectively. The stretching vibration peaks of P=O bonds are 1258 cm^−1^ and 1241 cm^−1^, and the asymmetric stretching vibration peak of P−O−C is 1045 cm^−1^ [39,40]. The peaks at 817 cm^−1^ and 678 cm^−1^ correspond to the −CH_2_ in-plane wobble absorption peaks of UCPR and MCPR, respectively. Simultaneously, an enhanced absorption peak of 1137 cm^−1^ was found, which may be attributable to the stretching vibration peak of the C−N bond after the CP reaction with urea and melamine.

### 3.2. NMR Spectrum Analysis

**Figure 2:**
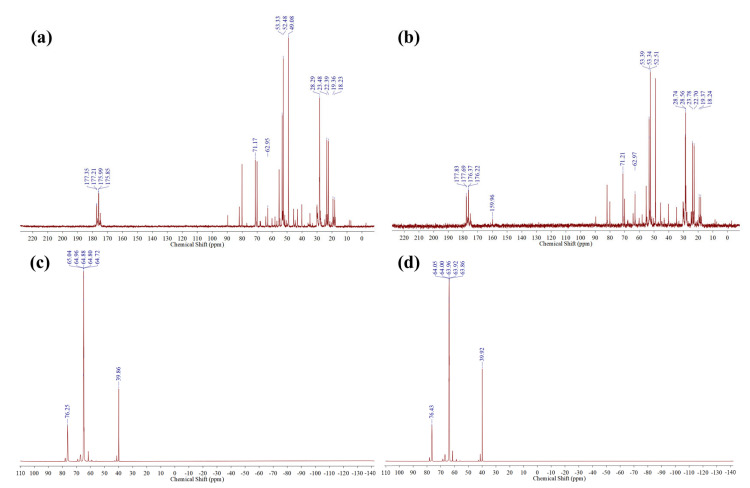
^13^C NMR spectra of (**a**) CP, (**b**) MCPR and ^31^P NMR spectra of (**c**) CP and (**d**) MCPR.

Figure 2a,b shows the ^13^C NMR spectral analysis of CP and MCPR. The chemical peak near 177.69 ppm corresponds to the −C=O bond of the amide, and the peak near 71.17 ppm corresponds to the alcohol group in the structure of CP. The chemical peaks near 62.95 ppm are attributed to the −N−C−N-group of the sec-carbon and the chemical peak near 53.34 ppm is attributed to the methoxy group, while the chemical peak near 28.56 ppm may be attributed to the −P−C−C− bond of the secondary carbon and the peak near 19.37 ppm may be attributed to the methyl carbon group. The chemical peak appearing in MCPR at 159.96 ppm is attributed to the methylimine group, indicating that CP reacts with MCPR. Referring to the ^31^P NMR spectrum of CP, MCPR also showed characteristic peaks at the same position, indicating the successful synthesis of MCPR (Figure 2c,d).

### 3.3. GPC

**Figure 3:**
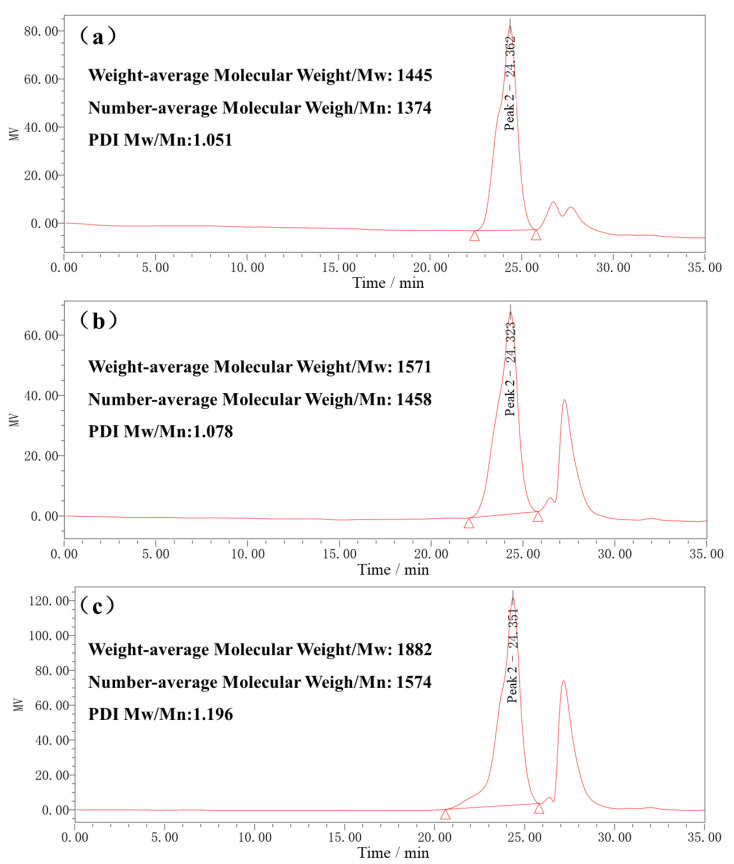
Automatic scale chromatogram of (**a**) CP (**b**) UCPR (**c**) MCPR.

As can be seen in Figure 3, the weight-average molecular weight Mw of UCPR is 126 higher than that of CP, and the number-average molecular weight Mn of UCPR is 84 higher than that of CP, which indicates that UCPR is a new phosphorus-nitrogen flame retardant generated by the reaction of CP and urea. The molecular weight distribution index Mw/Mn of UCPR increased less than that of CP, indicating that the synthesized polymer still has a relatively uniform molecular weight. At the same time, the weight-average molecular weight (Mw) of MCPR is 437 higher than CP, and the number-average molecular weight (Mn) of MCPR is 200 higher than CP, which indicates that MCPR is the reaction of CP and melamine to form a new phosphorus-nitrogen flame retardant. The molecular weight distribution index Mw/Mn of MCPR increased more relative to CP, indicating that the molecular weight distribution of the synthesized MCPR was wider and the degree of polydispersity increased.

### 3.4. Weigh Gain Rate

**Figure 4:**
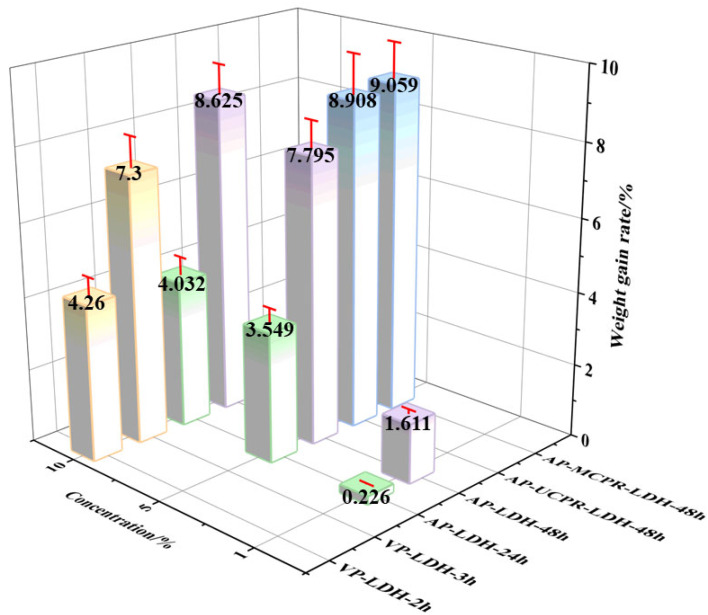
Average weight-gain rates of poplar veneer for different processes (VP: vacuum pressurized, AP: atmospheric pressure).

The concentration of the flame retardant suspension, the duration, and the impregnation process all influence the weight-gain rate of poplar veneer. The weight-gain rate of veneer specimens before and after impregnation was used to establish the optimal concentration and timing of impregnation. From the analysis of Figure 4, it can be seen that the differences in impregnation time and impregnating solution concentration have a greater impact on the weight gain of the specimens. The weight-gain rate of the veneer improved dramatically when vacuum-pressured impregnation at the same concentration was increased from 2 h to 3 h, but the process was more tedious. The increase of impregnation time from 24 h to 48 h at room temperature and pressure had a greater effect on the weight-gain rate, and the change of concentration from 5% to 10% had little effect on the weight-gain rate. Overall, when the poplar veneer specimens were impregnated for 48 h, the concentration of 5% flame retardant suspension was the better process, and the average impregnation weight-gain rates of LDH, UCPR-LDH, and MCPR-LDH poplar veneer were 7.795%, 8.908%, and 9.059%, respectively.

### 3.5. Cone Calorimetry

**Figure 5:**
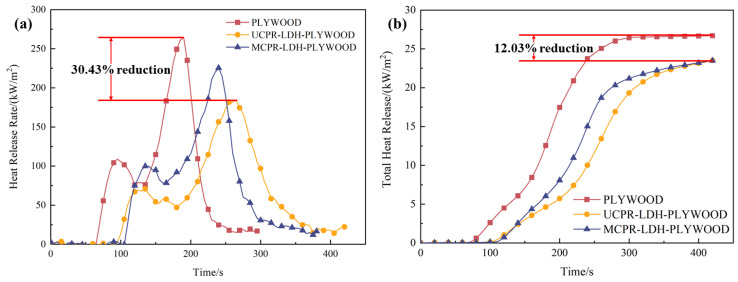
HRR (**a**), THR (**b**) plots of wood samples.

As can be observed in Figure 5a, there are two obvious exothermic peaks in the three layers of plywood [41,42]. The first exothermic peak of the control plywood was the earliest, and the first exothermic peak in flame-retardant plywood with UCPR-LDH and MCPR-LDH was lower and delayed compared to the control plywood. The above phenomenon indicates that LDH released a large amount of water and other gases, diluting the concentration of oxygen and combustible gases, when decomposed by heat, and absorbed heat to reduce the temperature of the burning surface of the material and slow down the rate of thermal degradation.

In the mid-burning period, the second exothermic peak is attributed to the charcoal layer on the burning surface. The flame either does not burn to the lower veneer or the veneer is not burned out, and a large stress is generated between the two, prompting the upper charcoal layer to fracture, which increases the flame contact area and accelerates the burning rate of plywood. Significantly, the second peak of the control plywood appeared earliest and the peak of heat release rate was highest, and the modified plywood showed different degrees of delayed heat release peak. Among the samples, the HRR of the UCPR-LDH flame-retardant plywood was reduced by 30.43% compared with the control group. This is because high temperature removes the OH- and ${\mathrm{CO}}_{3}^{2-}$ in the interlayer structure of LDH, fundamentally altering the structure. The LDH of the lamellar structure collapses and forms MgO and Al_2_O_3_ with better thermal stability, which can play the role of heat insulation and act as an oxygen barrier, thus reducing the burning rate of wood and delaying the appearance of the exothermic peak.

In Figure 5b, the THR at a burning time of 200 s was arranged by size as follows: control group > MCPR-LDH > UCPR-LDH plywood. The THR of UCPR-LDH plywood was lower than 8 kW/m^2^ at this time, and the THR of plywood modified by LDH and P-N FRs was significantly lower than that of the control group. The synergistic flame retardant effect was obvious, and the THR of modified UCPR-LDH and MCPR-LDH plywood was reduced by about 12.03% compared with that of the control group.

**Figure 6:**
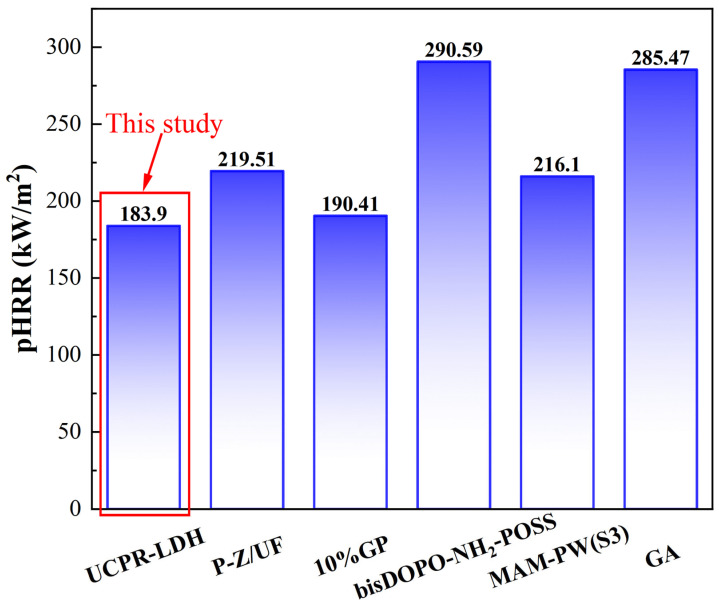
Comparison of flame-retardant plywood pHRR [43,44,45,46,47].

When comparing the peak heat release rate with those described in other literature on flame-retardant plywood [43,44,45,46,47], the results indicate that the majority of adhesives used in the literature were formaldehyde-based, which are not environmentally friendly. As depicted in Figure 6, the UCPR-LDH plywood developed in this study not only exhibits superior thermal barrier performance but also ensures formaldehyde-free emissions while being flame-retardant. This cost-effective and high-performance approach for producing flame-retardant plywood presents a novel concept for future industrial applications.

It is obvious from Table 3 that the TTI times of the modified plywood were all extended to different degrees. Among the modified plywood, the burning time of MCPR-LDH plywood was the longest, being improved by 42 s compared with the poplar control plywood, indicating that the specimens were not easily ignited and had good flame-retardant properties.

**Figure 7:**
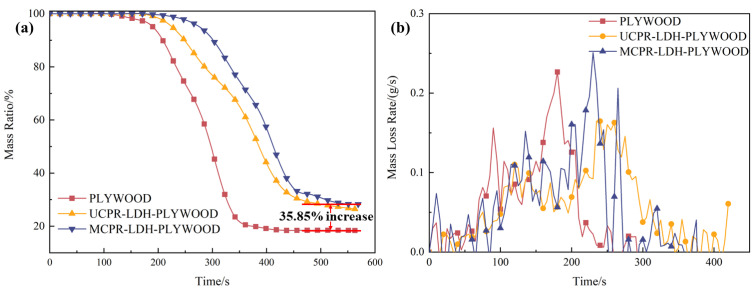
Mass ratio (**a**) and MLR (**b**) plots of wood samples.

As shown in Figure 7b, the trend of the MLR curve was similar to that of HRR and the peak positions of the two curves appeared the same, indicating that the mass loss rate and heat release rate showed a correlation trend. The thermal decomposition of plywood in the control group resulted in increased production of various combustible volatiles, and the heat release rate was elevated. At the same time, it also accelerated mass loss. According to Figure 7a, at about 560 s of combustion, the residual char rate of the control plywood was 18.28%, while the residual char rates of UCPR-LDH and MCPR-LDH were 26.20% and 28.49%, respectively. The char formation effect of plywood wood treated with LDH and P-N FRs was better. This indicates that LDH can catalyze char formation.

**Figure 8:**
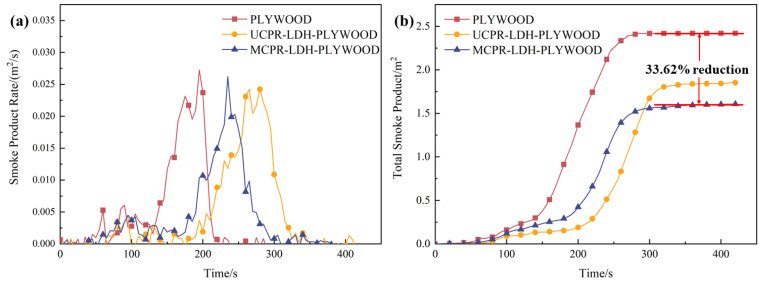
SPR (**a**) and TSP (**b**) plots of wood samples.

Figure 8a, showing the SPR curve of plywood, illustrates that its trend is similar to the HRR and MLR curves, indicating that heat release, mass loss and smoke release occur simultaneously during the combustion of plywood. The first peak produced by three-layer plywood at the beginning of ignition is attributed to the release of smoke, water vapor, and combustible volatiles from decomposition when heated. In the middle of combustion, the second peak is caused by continuous burning, which leads to the collapse and cracking of plywood and the release of volatile compounds resulting from incomplete combustion. The peaks of the modified plywood are all shifted back, indicating that the modified plywood has smoke suppression properties. In Figure 8b, the total smoke emission of the modified plywood was reduced compared with the control group, and the lowest smoke emission of MCPR-LDH plywood was 1.61 m^2^, which was 33.62% lower than that of the control plywood.

**Figure 9:**
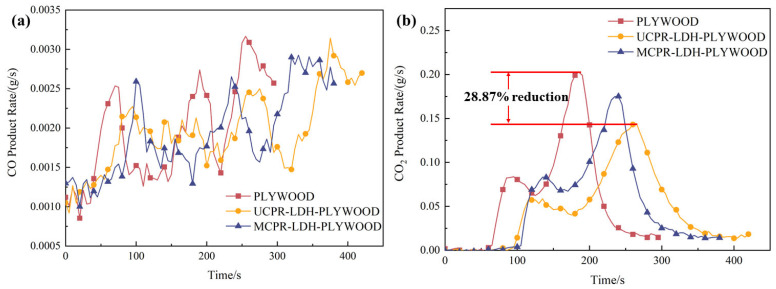
CO production rate (**a**), CO_2_ production rate (**b**) plots of wood samples.

Figure 9a,b shows graphs of CO and CO_2_ release rates. The CO_2_ release rate of UCPR-LDH and MCPR-LDH treated with flame retardant showed a decreasing trend compared with the control plywood. It is noteworthy that the peak CO_2_ release rate of UCPR-LDH plywood decreased by 28.87% compared with the control plywood. The modified plywood can promote the formation of carbon and reduce the production of combustible volatiles, thus reducing CO_2_ production. At the same time, the CO release rate of plywood treated with LDH and phosphorus-nitrogen flame retardant was lower than that of the control plywood, which could effectively reduce the concentration of carbon monoxide and the occurrence of carbon monoxide poisoning in case of fire and facilitate emergency evacuation, thus reducing casualties.

### 3.6. FTIR of Residual Carbon

**Figure 10:**
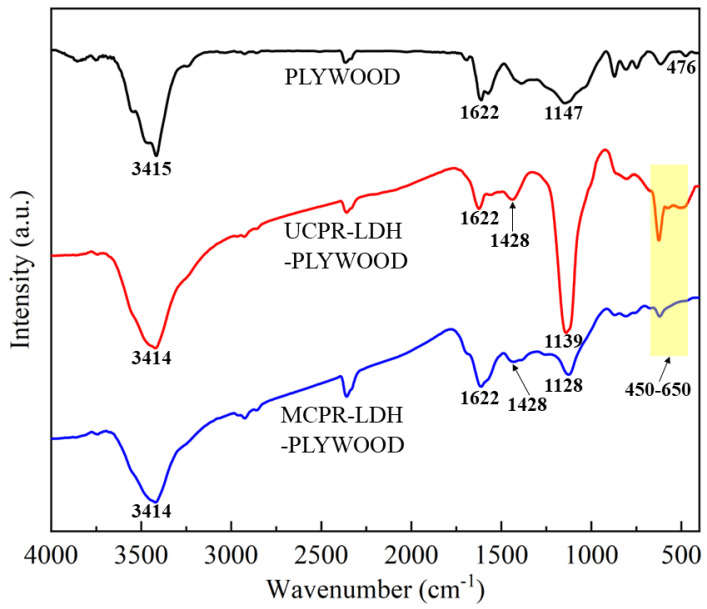
FTIR spectra of cone calorimetric residual carbon.

The residual carbon FTIR spectra of the control plywood, UCPR-LDH plywood, and MCPR-LDH plywood after cone calorimetry tests are presented in Figure 10. The broad peak near 3415 cm^−1^ represents the stretching vibration of O−H, N−H and interlayer water molecules, and the peak near 1622 cm^−1^ is the absorption peak of the stretching vibration of the C=C structure with a strong peak. Near 1139 cm^−1^ may be the stretching vibration peak of C−O. Meanwhile, the peak at 476 cm^−1^ observed in the control residual carbon may be attributed to the infrared absorption peak of Ca−O, because the inorganic filler chosen for the control plywood adhesive is calcium chloride; however, the disappearance of Ca^2+^ in UCPR-LDH and MCPR-LDH may be due to the substitution of Ca^2+^ by some other ions, such as ions with small radius Mg^2+^ small relative masses Al^3+^, Na^+^, etc. Compared with the control group, the peaks between 450−650 cm^−1^ in the modified plywood residual carbon may be attributed to the lattice vibrations of Mg−O, Al−O, and Mg−O−Al [48]. The peaks near 1428 cm^−1^ may be the peaks of antisymmetric stretching vibrations of the unreacted ${\mathrm{CO}}_{3}^{2-}$ in LDH or the stretching vibrations of P=O.

## 4. Conclusions

In summary, this study has demonstrated a cost-effective and simple approach for the preparation of formaldehyde-free plywood with flame-retardant properties. Two novel phosphorus-nitrogen flame retardants were successfully synthesized, with the optimal synthesis process of UCPR being a 4.55:1 molar ratio of CP and urea, with a reaction temperature and reaction time of 75 °C and 75 min. The optimal synthesis process of MCPR is a 9:1 molar ratio of CP and melamine, with a reaction temperature and reaction time of 70 °C and 75 min. Additionally, LDH was introduced and modified by intercalation to achieve a synergistic organic-inorganic flame retardance effect and enhance the flame-retardant efficiency of plywood. The flame-retardant formaldehyde-free plywood was produced through impregnation with a 5% concentration of flame retardant at room temperature and pressure for 48 h, as well as the substitution of inorganic filler components in the API adhesive with the above prepared flame retardants.

FTIR analysis revealed that the IR spectra of UCPR and MCPR were analogous to those of CP, with a discernible P-O-C bond at 1045 cm^−1^. NMR patterns showed that both synthesized UCPR and MCPR contained characteristic peaks of the corresponding groups. GPC chromatography demonstrated an increase in molecular weight for both UCPR and MCPR, as well as a more uniform distribution. The cone calorimetry test revealed that the UCPR-LDH plywood exhibited a total heat release of less than 8 kW/m^2^ at 200 s, and its pHRR was 30.43% lower than that of the control plywood, indicating a better thermal barrier performance. Meanwhile, the MCPR-LDH plywood demonstrated enhanced smoke suppression capabilities, with a reduction in TSP by 33.62% compared to the control plywood. The production of both CO and CO_2_ of the fire-retardant modified plywoods was reduced while effectively controlling the generation of smoke toxicity. FTIR analysis of the residual carbon obtained from the conical calorimetric test revealed the presence of Mg-O, Al-O and Mg-O-Al bonds, thus demonstrating the synergistic organic-inorganic flame-retardant effect of the plywood treated with flame retardants.

## Figures and Tables

**Table 1:** UCPR synthesis process.

Sample Code	CP Concentration	CP/Urea Molar Ratio	Temperature/°C	Time/min	Status
1	80%	0.91:1	135	120	White viscous gel
2	80%	1.82:1	135	120	White viscous gel
3	80%	4.55:1	70	30	Pale white translucent liquid
4	80%	4.55:1	75	30	Pale white translucent liquid, high viscosity
5	80%	4.55:1	80	30	Pale white translucent liquid, high viscosity
6	80%	4.55:1	75	120	White viscous gel
7	80%	1.82:1	75	120	White viscous gel
8	80%	4.55:1	75	75	White viscous liquid
9	65%	4.55:1	75	75	White liquid

Notice: Sample Codes 8 and 9 are for adding the reaction in batches.

**Table 2:** MCPR synthesis process.

Sample Code	CP Concentration	CP/MEL Molar Ratio	Temperature/°C	Time/min	Status
1	50%	9:1	70	60	Light white, not fully reacted
2	50%	12:1	70	60	Translucent, layered overnight
3	50%	6:1	70	75	Pale white, not completely reacted
4	50%	9:1	70	75	Translucent, viscosity too low
5	65%	6:1	70	75	Transparent liquid, with suspended particles
6	65%	9:1	70	75	Clear liquid, completely reacted
7	65%	12:1	70	75	Clear liquid, viscosity too low
8	65%	9:1	75	75	Translucent, layered overnight

Notice: Sample Codes 3, 4, 5, 6, 7, and 8 are for adding the reaction in batches.

**Table 3:** Results of cone calorimeter test for unmodified and modified plywood (UCPR-LDH and MCPR-LDH).

Sample	TTI (s)	THR (MJ/m^2^)	pHRR (kW/m^2^)	pCOPR (g/s)	pCO_2_ PR(g/s)	TSP (m^2^)	pSPR (m^2^/s)	MR (%)
PLYWOOD	63	26.69	264.35	0.00317	0.20274	2.42	0.02725	18.28
UCPR-LDH-PLYWOOD	97	23.51	183.90	0.00314	0.14420	1.85	0.02622	26.20
MCPR-LDH-PLYWOOD	105	23.48	225.45	0.00293	0.17477	1.61	0.02426	28.49

## Data Availability

Not applicable.
